# Visit-to-visit systolic blood pressure variability in patients with ST-elevation myocardial infarction predicts long-term cardiovascular outcomes

**DOI:** 10.1038/s41371-019-0176-0

**Published:** 2019-02-18

**Authors:** Moon-Seung Soh, Jin-Sun Park, Kyoung-Woo Seo, Hyoung-Mo Yang, Hong-Seok Lim, Byoung-Joo Choi, So-Yeon Choi, Myeong-Ho Yoon, Gyo-Seung Hwang, Seung-Jea Tahk, Joon-Han Shin

**Affiliations:** Department of Cardiology, Ajou University School of Medicine, Suwon, South Korea

**Keywords:** Clinical trials, Prognosis, Myocardial infarction, Hypertension, Preventive medicine

## Abstract

Elevated visit-to-visit blood pressure variability (BPV), independent of mean BP, has been associated with cardiovascular events. However, its impact after ST-elevation myocardial infarction (STEMI) has not been established. This study aimed to investigate the prognostic impact of BPV on patients after STEMI. We analyzed the data and clinical outcomes of STEMI survivors who underwent successful primary coronary intervention from 2003 to 2009. BP was measured at discharge and at 1, 3, 6, 12, 24, and 36 months, and we calculated BPV as the intra-individual standard deviations (SDs) of systolic BP (SBP) across these measurements. We classified the patients as high and low-BPV group, and evaluated the outcomes: occurrence of major adverse cardiovascular events (MACEs), death, recurrent myocardial infarction, and target vessel revascularization within 60 months. We enrolled 343 patients, and mean follow-up duration was 68 ± 34 months (median: 76 months). Mean and median SBP SDs were 13.2 and 12.3 mmHg, and patients were divided into one of the two groups based on the median (high-BPV group = SD ≥ 12.3 mmHg; low-BPV group = SD < 12.3 mmHg). The MACE-free survival in the high-BPV group was significantly worse than that in low-BPV group (log-rank *p* = 0.035). For the high-BPV group, the risk of a MACE significantly increased by 57% (95% confidence interval: 1.03–2.39; *p* = 0.038). Visit-to-visit systolic BPV was associated with increased rates of adverse clinical outcomes in patients after STEMI. Careful assessment of BP and attempts to reduce BPV might be also important in STEMI survivors.

## Introduction

Hypertension (HTN) is the most important risk factor for a variety of vascular events [1, 2], but independent of mean blood pressure (BP), blood pressure variability (BPV) has been associated with clinical cardiovascular events in some diseases [3–8]. BPV is known to be the result of complex interactions between extrinsic environmental and behavioral factors and intrinsic cardiovascular regulatory mechanisms such as neural reflexes and humoral influences that are not yet completely understood. BPV can be classified into very short-term, short-term, mid-term, and long-term BPV. Long-term BPV can lead to subclinical organ damage, poor renal function, all-cause mortality, and also cardiovascular events [3]. Visit-to-visit BPV, a kind of long-term BPV, was an important predictor of cardiovascular events in some large trial studies [4–6].

A sub-study of the Anglo-Scandinavian Cardiac Outcomes Trial Blood Pressure Lowering Arm (ASCOT-BPLA) reported that visit-to-visit BPV in hypertensive patients increased the risk of acute coronary events. The authors used standard deviation (SD) and variation independent of mean 24-h ambulatory BP monitoring as BPV indices [4]. Authors of a sub-study of the Ongoing Telmisartan Alone and in Combination with Ramipril Global Endpoint Trial (ONTARGET) found that myocardial infarction (MI) risk was affected by visit-to-visit systolic blood pressure (SBP) changes from baseline in coronary artery patients [5]. In addition, increased visit-to-visit systolic BPV (based on SD) in hypertensive patients led to increased cardiovascular disease and mortality at follow-up in a sub-study of the Antihypertensive and Lipid-Lowering Treatment to Prevent Heart Attack Trial (ALLHAT) [6].

There are several studies about the prognostic impact of visit-to-visit BPV in hypertensive and coronary artery patients [4–6], and ST-elevation myocardial infarction (STEMI) is a serious cardiovascular event. However, the impact of visit-to-visit BPV after STEMI has not yet been established. In this study, we aimed to investigate the prognostic impact of BPV on STEMI patients with successful primary coronary intervention.

## Methods

### Study population

We analyzed the retrospective data and clinical outcomes of STEMI survivors who underwent successful primary coronary intervention from 2003 to 2007 in a single center, the Ajou University School of Medicine in Suwon, South Korea. We defined STEMI as new ST elevation at the J point in at least 2 contiguous leads of ≥ 2 mm (0.2 mV) in men or ≥ 1.5 mm (0.15 mV) in women in leads V2–V3 and/or of ≥ 1 mm (0.1 mV) in other contiguous chest leads or the limb leads from the Task Force for the Universal Definition of Myocardial Infarction [9]. New or presumed new left bundle branch block has also been considered equivalent to STEMI [10].

**Fig. 1:**
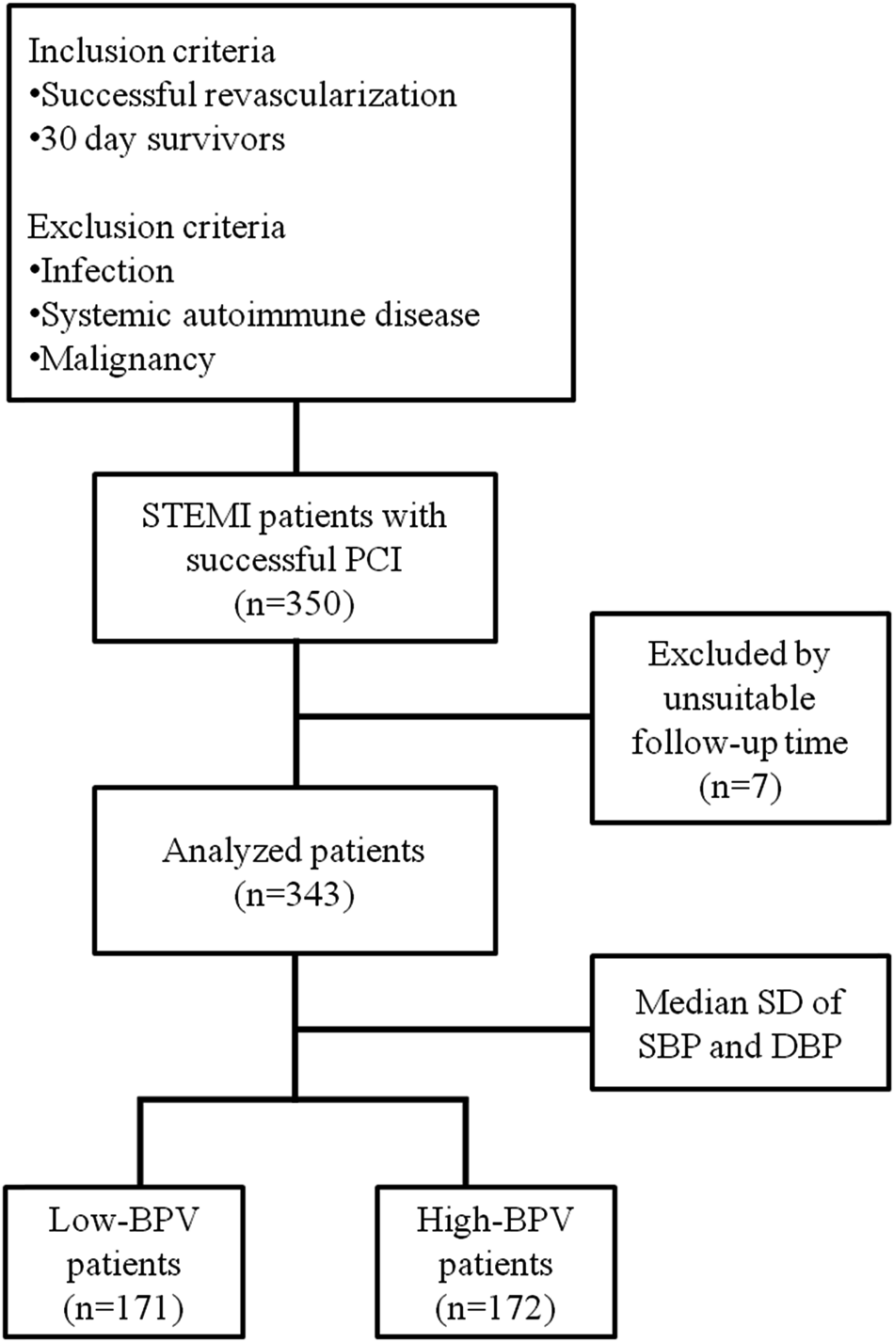
Study population. STEMI ST elevation myocardial infarction, PCI percutaneous coronary intervention, SD standard deviation, SBP systolic blood pressure, DBP diastolic blood pressure, BPV blood pressure variability

The inclusion criteria were successful revascularization and 30-day survival after STEMI, and we defined successful revascularization as Thrombolysis In Myocardial Infarction (TIMI) grade 3 flow and residual stenosis in the infarct-related artery < 30%. We excluded patients with active inflammation whose various conditions could have affected BPV, such as infection, systemic autoimmune disease, and malignancy (Fig. 1). Finally, we collected 350 patients’ data and excluded 7 patients who had unsuitable follow-up times for analysis. All participants provided informed consent, and the study was approved by the institutional review board (IRB: AJIRB-MED-MDB-17-402).

### Measurement of BPV

**Fig. 2:**
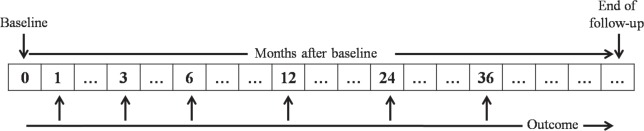
Visit-to-visit BP assessment. BP blood pressure, SBP systolic blood pressure, DBP diastolic blood pressure

We estimated BPV by measuring SBP and diastolic blood pressure (DBP) on admission and at out-patient follow-up, and we measured BP at discharge and at 1, 3, 6, 12, 24, and 36 months (Fig. 2). We defined BPV as the intra-individual SD of BP across the visits. We used office BP measurement (OBPM) by automated validated device (HEM-7080 IC model, OMRON Health Care, Kyoto, Japan). BP was checked in all participants after 20 min at rest in a sitting position. We then classified the patients as having high or low-BPV based on the median SD.

### Clinical outcomes

Our primary outcomes of interest were all-cause mortality, recurrent MI, target vessel revascularization (TVR), and the composite, major adverse cardiovascular events (MACEs), and we assessed these outcomes during 5 years after the start of the study. We excluded participants when we lost them to follow-up and after the end of the analysis period (January 31, 2010). We identified MI, TVR, and mortality from the patients’ National Health Insurance service data and/or telephone interviews with patients or their families, and we confirmed the data by physician adjudication using medical and hospital records, in-person examinations, electrocardiograms, angiographies, echocardiograms, and laboratory or imaging studies. We defined recurrent MI based on the universal definition [9], and we defined TVR as any repeat percutaneous revascularization or surgical bypass of the original target lesion site that occurred 30 or more days after percutaneous coronary intervention (PCI), driven by clinical findings (presence of ischemic symptoms and/or a positive functional ischemia study), in the presence of a diameter stenosis ≥ 50% as determined by coronary angiography. In the absence of ischemic symptoms or a positive functional ischemia study, we also considered revascularization for a diameter stenosis ≥ 70% be clinically driven.

### Covariables

We collected the covariable data, age, sex, and body mass index (BMI) at first admission, calculating BMI as weight (kg)/height^2^ (m^2^); we also defined old age as > 65 years. We obtained the patients’ medical histories, including HTN, diabetes mellitus, dyslipidemia, previous cerebrovascular accident and current and past smoking, from their medical records and considered patients who had a current SBP/DBP ≥ 140/90 mmHg or who were receiving antihypertensive therapy to have HTN. We based diabetes mellitus on any of the following criteria: use of insulin or oral hypoglycemic agents, fasting glucose ≥ 126 mg/dL, or non-fasting glucose ≥ 200 mg/dL. We defined dyslipidemia as low-density lipoprotein cholesterol (LDL-C) ≥ 140 mg/dL and/or triglycerides (TG) ≥ 150 mg/dL, high-density lipoprotein cholesterol (HDL-C) < 40 mg/dL, or receiving lipid-lowering therapy. For family history, we looked at a first-degree male relative (e.g., father, brother) who had suffered from a coronary heart disease before the age of 55 or a first-degree female relative who had suffered from one before age 65.

We based antihypertensive medication and statin use on medication inventories and interviews conducted at discharge from first admission after STEMI with PCI, and the medications we studied were angiotensin-converting enzyme inhibitors (ACEi), angiotensin II receptor blockers (ARB), beta blockers, and calcium channel blockers (CCB).

We investigated infarct-related lesions, number of stenotic coronary vessels, and the type of deployed stent (bare metal or drug-eluting) by coronary angiography at the time of the PCI. We assessed left ventricular end diastolic and systolic diameter, left ventricular ejection fraction (LVEF), wall motion score index, and significant mitral regurgitation (MR) from echocardiographic findings, and we defined significant MR as ≥ grade 3.

### Statistical analysis

We used SPSS 13.0 statistical software package (SPSS, Chicago, IL, USA) for all calculations. We compared the patients with high versus low-BPV and used the log-rank test to draw Kaplan–Meier survival curves for the primary outcomes (all-cause mortality, recurrent MI, TVR, MACEs). We used Cox proportional hazard models to estimate the adjusted hazard ratios (HRs) of the high-BPV group for the risk of the primary outcomes based on the intra-individual SDs of BP across the visits by age, gender, HTN, diabetes mellitus, dyslipidemia, smoking, Killip class, LVEF, and BPV  (≥ 12.3 mmHg). Finally, we calculated the multivariable-adjusted HRs for each outcome associated with BPV using multivariable logistic regression analysis. We considered *p* < 0.05 to be significant.

## Results

We enrolled 343 patients (276 males, 67 females). Their baseline clinical characteristics are presented in Table 1. The mean age of the study participants was 58 ± 12 years, and the mean follow-up duration was 68 ± 34 months (median: 76 months). The intra-individual mean SDs for SBP and DBP were 13.2 ± 7.6 and 8.9 ± 4.4 mmHg, respectively, and the intra-individual median SDs for each were 12.3 and 8.6 mmHg, also respectively. As we noted above, we classified all patients as high (SD ≥ 12.3 mmHg) and low-BPV (SD < 12.3 mmHg) group. We found no statistically significant results for any end points including MACEs based on diastolic BPV (HR: 0.166; 95% confidence interval [CI]: −0.093 to 0.468; *p* = 0.189).

**Table 1:** Baseline characteristics by systolic BPV group

	Characteristics	Low-BPV (*n* = 171)	High-BPV (*n* = 172)	*p*-Value
Clinical characteristic	Male, *n* (%)	136 (80)	134 (78)	0.714
	Age (year-old)	58 ± 13	59 ± 12	0.731
	Old age (> 65 years)	51 (30%)	57 (33%)	0.510
	BMI (kg/m^2^)	24 ± 5	25 ± 5	0.783
Medical history	Hypertension, *n* (%)	53 (31)	78 (45)	0.006
	Diabetes mellitus, *n* (%)	34 (20)	41 (24)	0.377
	Dyslipidemia, *n* (%)	12 (7)	11 (6)	0.818
	Previous CVA, *n* (%)	3 (2)	5 (3)	0.481
	Familial history, *n* (%)	6 (3.5)	12 (7.0)	0.150
	Smoking, *n* (%)	114 (66.7)	117 (68)	0.790
Medication	ACEi, *n* (%)	100 (59)	116 (67)	0.086
	ARB, *n* (%)	52 (30)	54 (31)	0.844
	Beta blocker, *n* (%)	114 (67)	99 (58)	0.083
	CCB, *n* (%)	32 (19)	46 (27)	0.076
	Statin, *n* (%)	90 (53)	94 (55)	0.709
Infarction related lesion	LAD, *n* (%)	100 (59)	89 (52)	0.211
	LCX, *n* (%)	15 (9)	18 (11)	0.596
	RCA, *n* (%)	56 (33)	65 (38)	0.33
Number of stenotic coronary vessels	1 Vessel disease, *n* (%)	77 (45)	72 (42)	0.555
	2 Vessel disease, *n* (%)	51 (30)	60 (35)	0.318
	3 Vessel disease, *n* (%)	43 (25)	40 (23)	0.684
Type of deployed stent	Bare metal stent, *n* (%)	103 (60)	108 (63)	0.566
	Drug-eluting stent, *n* (%)	73 (43)	72 (42)	0.808
Echocardiographic findings	LVEDD (mm)	50 ± 9	50 ± 8	0.882
	LVESD (mm)	33 ± 8	34 ± 2	0.602
	LVEF (%)	53.4 ± 11.8	51.7 ± 11.9	0.167
	WMSI	1.39 ± 0.24	1.56 ± 0.37	0.581
	Significant MR (≥ Grade 3), *n* (%)	6 (4)	4 (2)	0.516

*BPV* blood pressure variability, *BMI* body mass index, *CVA* cerebrovascular accident, *ACEi* angiotensin-converting enzyme inhibitor, *ARB* angiotensin II receptor blocker, *CCB* calcium channel blocker, *LAD* left anterior descending artery, *LCX* left circumflex artery, *RCA* right coronary artery, *LVEDD* left ventricular end diastolic diameter, *LVESD* left ventricular end systolic diameter, *LVEF* left ventricular ejection fraction, *WMSI* wall motion score index, *MR* mitral regurgitation

There were no significant differences in baseline clinical characteristics, sex, age, and BMI, between the two BPV groups, and we also found no statistically significant differences in the patients’ medical histories, medications at discharge, or angiographic and echocardiographic findings. There were more hypertensive patients in the high systolic BPV group (45%) than in the low-BPV group (31%; *p* = 0.006).

**Fig. 3:**
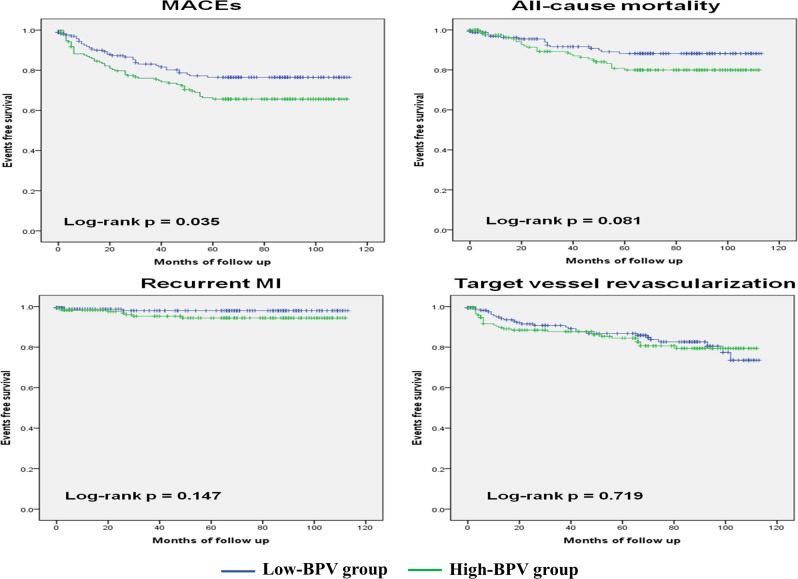
Kaplan–Meier survival curves for free of adverse outcomes in high systolic BPV group and low systolic BPV group. MACEs major adverse cardiovascular events, MI myocardial infarction

Kaplan–Meier survival curves showed that the MACE-free survival in the high-BPV group was significantly worse than that in the low-BPV group (*p* = 0.035, Fig. 3). For the other end points, all-cause mortality (*p* = 0.081), recurrent MI (*p* = 0.147), and TVR (*p* = 0.719), we also found no statistically significant results. Only all-cause mortality tended to be worse in the high-BPV group than in the low-BPV group over the follow-up months.

In univariate analysis of the clinical outcomes, the occurrence of MACEs was higher in the high systolic BPV group (38%) than in the low-BPV group (26%; *p* = 0.011), and all-cause mortality showed a higher tendency in the high-BPV group (16%) than in the low-BPV group (9%; *p* = 0.055). There were no statistically significant differences between the two systolic BPV groups in recurrent MI (*p* = 0.128) and TVR (*p* = 0.677). Table 2 shows the multivariable logistic regression analysis results for visit-to-visit systolic BPV for adverse outcomes. In the high systolic BPV group, the relative risk of MACEs was significantly higher (HR: 1.57; 95% CI: 1.03–2.39; *p* = 0.038). All-cause mortality showed a higher tendency in the high-BPV group, but the result was not statistically significant (HR: 1.72; 95% CI: 0.93–3.17; *p* = 0.085).

**Table 2:** Multivariable logistic regression analysis of the presence of visit-to-visit systolic blood pressure variability for adverse outcomes

Clinical outcomes	Hazard ratio	95% CI	*p-*Value
MACEs	1.57	1.03–2.39	0.038
All-cause mortality	1.72	0.93–3.17	0.085
Recurrent MI	2.57	0.68–9.70	0.163
TVR	1.10	0.65–1.87	0.72

*MACEs* major adverse cardiovascular events, *MI* myocardial infarction, *TVR* target vessel revascularization, *CI* confidence interval

In Cox-regression analysis adjusted by the effects of the covariables, including mean SBP during follow-up period, for each primary outcome (Table 3), age and higher systolic BPV (≥ 12.3 mmHg) were significant independent predictors of the occurrence of MACE outcomes (HR: 1.039; 95% CI: 1.016–1.061; *p* = 0.001; HR: 1.722; 95% CI: 1.083–2.738; *p* = 0.022). For all-cause mortality, age, diabetes mellitus, and LVEF were significant independent predictors for the adverse outcome. All-cause mortality and recurrent MI showed a higher tendency in the high systolic BPV group (≥ 12.3 mmHg), but the result was not statistically significant (HR: 1.734; 95% CI: 0.936–3.214; *p* = 0.08; HR: 2.308; 95% CI: 0.9–5.917; *p* = 0.082). There were no independent predictors of recurrent MI and TVR, the other primary outcomes.

**Table 3:** Cox-regression analysis of the presence of visit-to-visit systolic blood pressure variability for adverse outcomes

Variables	MACEs			All-cause mortality			Recurrent MI			TVR		
	Adjusted HR	95% CI	*p*-Value	Adjusted HR	95% CI	*p*-Value	Adjusted HR	95% CI	*p*-Value	Adjusted HR	95% CI	*p*-Value
**Age**	1.039	1.016–1.061	0.001	1.095	1.061–1.131	<0.001	0.993	0.955–1.034	0.749	0.987	0.961–1.014	0.339
**Gender**	1.038	0.53–2.035	0.913	0.957	0.429–2.137	0.915	0.619	0.163–2.347	0.48	1.242	0.494–3.123	0.645
**Mean SBP during follow-up**	1.003	0.995–1.01	0.51	1.004	0.993–1.015	0.476	0.998	0.978–1.018	0.856	1.002	0.993–1.01	0.661
**Diabetes mellitus**	1.612	0.933–2.786	0.087	2.047	1.043–4.017	0.037	1.018	0.347–2.986	0.974	1.3	0.657–2.57	0.451
**Dyslipidemia**	0.562	0.202–1.566	0.271	3.184	0.631–16.075	0.161	0.605	0.074–4.948	0.639	1.087	0.346–3.42	0.886
**Smoking**	1.379	0.769–2.471	0.28	1.654	0.783–3.493	0.187	1.37	0.41–4.576	0.609	1.081	0.512–2.284	0.837
**Killip classification**	1.021	0.776–1.342	0.884	1.004	0.723–1.396	0.98	0.289	0.083–1.007	0.051	1.098	0.772–1.562	0.603
**LVEF**	0.98	0.959–1.0	0.053	0.958	0.932–0.986	0.004	0.992	0.952–1.033	0.697	1.013	0.987–1.038	0.335
**BPV** **≥** **12.3** **mmHg**	1.722	1.083–2.738	0.022	1.734	0.936–3.214	0.08	2.308	0.9–5.917	0.082	1.201	0.671–2.15	0.538

*MACEs* major adverse cardiovascular events, *SBP* systolic blood pressure, *LVEF* left ventricular ejection fraction, *BPV* blood pressure variability, *MI* myocardial infarction, *TVR* target vessel revascularization, *CI* confidence interval, *E7* 10^7^

## Discussion

The present study demonstrated for the first time the close relationship between visit-to-visit systolic BPV and long-term cardiovascular outcomes in patients with STEMI who underwent successful PCI.

Many observational studies have shown the relationships between systolic BPV and mortality, coronary heart disease, stroke, and white matter disease [7, 8, 11–14]. Increased visit-to-visit BPV can attenuate hemodynamic homeostasis, cause end-organ damage, and have negative impacts on the vascular system, leading to mortality [15, 16]. Studies have suggested a number of potential mechanisms for long-term BPV, particularly increased arterial stiffness [17, 18], subclinical inflammation [19], and endothelial dysfunction [15], and high visit-to-visit BPV might reflect the lower artery elasticity with functional changes in the large vessels [20]. The pathophysiology of STEMI is most often from coronary thrombosis after plaque rupture in a major coronary artery that had been previously affected by atherosclerosis. Stiffness and endothelial function of the coronary artery can affect STEMI, with inflammatory cascades as well.

The intra-individual mean (median) SDs of SBP and DBP were 13.2 ± 7.6 (12.3) mmHg and 8.9 ± 4.4 (8.6) mmHg, respectively. In the 2010 ASCOT-BPLA sub-study, visit-to-visit BPV increased the risk of acute coronary events; the mean SDs for SBP and DBP were 10.99 and 6.26 mmHg, respectively, in an amlodipine-based regimen and 13.42 and 6.98 mmHg, also respectively, in an atenolol-based regimen [4]. In a sub-study of the Ohasama Study (day-by-day BPV), the median SDs for SBP and DBP were 8.6 and 6.9 mmHg, respectively [21]. In Wu et al. [22], the median SDs of SBP and SBP were 8.53 and 4.98 mmHg, respectively, and in Gondo et al. [23], the median SDs were 10.2 and 6.9 mmHg, also respectively. We identified more BPV in our study than other studies have found, and only had similar values to those from the ASCOT-BPLA atenolol regimen. We concluded that patients with STEMI, in particular, had higher BPV than the patients with other cardiovascular diseases, emphasizing the importance of the regulating BPV in STEMI.

BPV can be classified by the duration of measurement [3]. Very short-term (beat-to-beat) and short-term (24-h) BPV are affected by humoral factors such as the sympathetic drive and cardiopulmonary reflex, so these can change easily. Visit-to-visit BPV might not only consist of spontaneous BP variations, but also reflect the poor BP and cardiovascular control physiologically, in treated patients and inconsistent OBPM during follow-up [24, 25]. Further, the differences between the two BPV groups in adverse outcomes based on the Kaplan–Meier survival curves increased over the 60-month follow-up period, reflecting the value of visit-to- visit BPV for long-term prognosis of STEMI with PCI.

In the study analysis by diastolic BPV, we obtained no statistically significant results for all end points including MACEs. Some BPV studies have also not shown significant results for diastolic BPV [4, 6, 24], but other studies have found different results. Wu et al. reported that visit-to-visit diastolic BPV could be a risk factor for mortality in the elderly [22]. Kikuya et al., in the Ohasama Study sub-study, showed that the Kaplan–Meier survival curve by SD of DBP (day-by-day BPV) for cardiovascular mortality and mortality changed across quartiles of SD distributions [21]. It has been suggested that increased visit-to-visit BPV is associated with diastolic dysfunction [26], and that low DBP and large decreases in DBP could lead to decreased organ perfusion, especially the heart, which is only perfused during diastole [27]. In our study, most of the participants already lacked coronary function because of the STEMI, which might have affected our results. We can conclude that systolic BPV has greater prognostic impact for adverse outcomes in STEMI, but additional research is needed to investigate the difference between systolic and diastolic BPV for cardiovascular risk.

There are several limitations in this study. We investigated relatively few people (*n* = 343) and conducted a retrospective study at a single center, and thus, large, multicenter cohort studies will be needed in the future. The effects of antihypertensive medications and statins over time also could have affected our results. There were no differences in the kinds of medications on discharge of first admission between the high and low-BPV groups, but the quantities or doses of each medication could not be considered. Especially, the patients on higher doses of anti-hypertensive medications could have higher BPV. Furthermore, the types and doses could have changed between follow-up months. To regulate and reduce BPV, the effects of antihypertensive medications should be compared, but we did not do this in our study. Some studies on how antihypertensive agents affect BPV suggest better possibility of CCBs and long-acting ARBs compared with other drugs [28–30]. Extended studies should control antihypertensive medications over time in details, including types and doses, and also compare the effects of each medication for BPV regulation.

In this study, visit-to-visit SBP variability was associated with increased rates of adverse clinical outcomes in patients after STEMI with PCI. Thus, careful assessment of BP and attempts to reduce BPV might also be important for STEMI.
